# Inhaled Corticosteroids and Non-Tuberculous Mycobacteria Risk in Patients with COPD

**DOI:** 10.3390/jcm15093352

**Published:** 2026-04-28

**Authors:** Emma Moerk Borremose, Victor Naestholt Dahl, Anna Kubel Vognsen, Troels Lillebaek, Andreas Fløe, Tor Biering-Sørensen, Barbara Bonnesen, Josefin Eklöf, Pradeesh Sivapalan, Jens-Ulrik Stæhr Jensen

**Affiliations:** 1 Copenhagen Respiratory Research, Department of Medicine, Herlev and Gentofte Hospital, University of Copenhagen, 2900 Hellerup, Denmarkanna.kubel.vognsen.01@regionh.dk (A.K.V.); jens.ulrik.jensen@regionh.dk (J.-U.S.J.); 2 Department of Infectious Diseases, Aarhus University Hospital, 8200 Aarhus, Denmark; 3 International Reference Laboratory of Mycobacteriology, Statens Serum Institut, 2300 Copenhagen, Denmark; 4 Global Health Section, Department of Public Health, University of Copenhagen, 1353 Copenhagen, Denmark; 5 Department of Respiratory Diseases and Allergy, Aarhus University Hospital, 8200 Aarhus, Denmark; 6 Department of Cardiology, Herlev and Gentofte Hospital, University of Copenhagen, 2900 Hellerup, Denmark; 7 Center for Translational Cardiology and Pragmatic Randomized Trials, Department of Biomedical Sciences, Faculty of Health and Medical Sciences, University of Copenhagen, 2900 Hellerup, Denmark; 8 Department of Cardiology, Copenhagen University Hospital Rigshospitalet, 2100 Copenhagen, Denmark; 9 Steno Diabetes Center Copenhagen, 2730 Herlev, Denmark; 10 Department of Clinical Medicine, Faculty of Health Sciences, University of Copenhagen, 2200 Copenhagen, Denmark

**Keywords:** chronic obstructive pulmonary disease, non-tuberculous mycobacteria, inhaled corticosteroids

## Abstract

**Background/Objectives**: Inhaled corticosteroids (ICS) increase the risk of pneumonia caused by various pathogens in patients with chronic obstructive pulmonary disease (COPD). Treatment may also increase the risk of infection with non-tuberculous mycobacteria (NTM), although evidence remains limited. The aim of this study was to assess the association between ICS treatment and the risk of NTM isolation among patients with COPD. **Methods**: This retrospective register-based cohort study included patients with a specialist-verified COPD diagnosis between 2008 and 2021. ICS exposure was based on redeemed prescriptions during the year preceding the index date. Exposure was calculated as the mean daily budesonide-equivalent dose and categorized as none, low, medium, or high. A cause-specific Cox proportional hazards regression model with death as a competing risk was applied, adjusted for potential confounders. Sensitivity analyses included, among others, an inverse probability of treatment weighted model, and a time-dependent Cox regression model. **Results**: A total of 120,006 patients were included, with a median follow-up time of 4.9 years. During follow-up, 378 (0.32%) patients reached the primary endpoint. Medium- and high-dose ICS were associated with an increased hazard of NTM isolation, with hazard ratios of 1.39 (95% CI 1.06–1.88, *p* = 0.020) and 1.52 (95% CI 1.14–2.04, *p* = 0.005), respectively. This association remained significant for high-dose ICS across all sensitivity analyses. **Conclusions**: In patients with COPD, ICS treatment was associated with an increased and dose-dependent hazard of NTM isolation, particularly at high doses. High-dose ICS should, therefore, be prescribed with caution.

## 1. Introduction

Globally, the incidence of non-tuberculous mycobacteria (NTM) has been rising, likely due to environmental changes, aging populations, increased use of immunosuppressive agents, and increasing prevalence of chronic lung disease, as well as improved diagnostic awareness [1,2]. However, non-tuberculous mycobacterial pulmonary disease (NTM-PD) may be substantially underreported, as symptoms of chronic obstructive pulmonary disease (COPD) often overlap with those of NTM-PD, and symptoms of NTM-PD are often attributed to deterioration of underlying structural disease, potentially leading to underdiagnosis [3]. NTM-PD imposes a considerable health burden, with direct healthcare costs estimated to be 4–4.8 times higher for cases compared to controls [4,5]. Treatment success is achieved in only about 60% of patients with *Mycobacterium avium* complex (MAC), the most common NTM species group, while adverse effects are frequent, and approximately one in six patients discontinue therapy [6]. The five-year mortality rate approaches 51% [7]. Identifying modifiable risk factors, such as inhaled corticosteroids (ICS), is therefore clinically important.

ICS is widely prescribed for the management of COPD. ICS treatment of COPD patients reduces the risk of exacerbations and is recommended as an add-on to long-acting bronchodilators in patients with a history of exacerbations, especially in a situation with eosinophilic inflammation [8,9]. While reducing the risk of exacerbations, ICS treatment comes with a price of increased risk of bacterial lung infection [10]. Therefore, both the indication for ICS treatment and the choice of dosage should be considered carefully. Few studies regarding the association between ICS and NTM have been performed, all with different study design, study groups, and exclusion criteria and with only one being a cohort study focusing solely on the association between NTM-PD and ICS use in patients with COPD [11,12,13,14,15]. Using Denmark’s comprehensive national health registers and the unique ability to link data across them, we have the possibility to examine the association between NTM and ICS exposure in a nationwide COPD population.

The aim of this nationwide cohort study was to assess the association between ICS use and the risk of NTM isolation among patients with COPD and to determine whether this association is dose-dependent. We hypothesize that there is a dose-dependent relationship between ICS treatment and hazard of NTM isolation.

## 2. Materials and Methods

### 2.1. Study Design and Study Population

We conducted a nationwide register-based retrospective cohort study using data from the Danish Register of Chronic Obstructive Pulmonary Disease (DrCOPD). DrCOPD was established in 2008 and is a nationwide register including all patients aged > 30 with a specialist-verified diagnosis of COPD, who are treated by a respiratory medicine specialist in a hospital setting. The database contains several variables including body mass index (BMI), tobacco exposure, and FEV1% [16]. Using a unique personal identification number, data were linked across national registers including the following: (1) the Danish Civil Registration System containing data on sex, date of birth, and vital status; (2) the Danish Register of Cause of Death holds data on date of death, cause of death, and circumstances surrounding death; (3) the Danish National Patient Register (DNPR) containing information on all hospital admissions since 1977 and all outpatient hospital visits since 1995. Each visit was coded with one primary and one or more secondary diagnoses according to the International Classification of Diseases. Since 1994, DNPR has registered all diagnosis according to ICD-10. (4) The Danish National Database of Reimbursed Prescriptions (DNDRP) holds information on all redeemed prescriptions dispensed by pharmacies and hospital-based outpatient pharmacies in Denmark since 2004, including data on dispensation, quantity, strength, and formulation. (5) The International Reference Laboratory of Mycobacteriology at Statens Serum Institut performs all mycobacterial diagnostics in Denmark and contributes nationwide microbiological data on all NTM isolated from respiratory samples [17,18,19,20,21].

The cohort included all patients registered in DrCOPD treated in a hospital setting between 1 January 2008 and 31 December 2021. The index date was defined as the first hospital outpatient visit or admission. Patients were excluded if they had diagnoses or medications indicating an increased risk of infection, including cystic fibrosis (DE84), congenital lung malformations (DQ33), immunodeficiency (DD80-89), malignant neoplasm except non-melanoma skin cancers (DC00-43, DC45-99), or use of immunosuppressive drugs (L04A). Patients with NTM isolates before the index date were excluded as well. Based on sample size calculation, the aim of this study was to have 8150 study participants per exposure group; see Supplementary Table S1 for calculations.

### 2.2. Exposure and Outcome

Prescriptions of ICS dispensed in the year before the index date were identified for monotherapy inhalers (R03BA01-02, 05-09), dual therapy inhalers (R03AK06-15), and triple therapy inhalers (R03AL08-09, 11-12). All prescriptions were converted into budesonide-equivalent doses; conversion rates are previously published [22] (see Supplementary Table S2). The mean daily dose within the year prior to the index date was calculated and categorized into four groups: (1) no ICS use, (2) low-dose ICS (<400 µg/day), medium-dose ICS (400–800 µg/day), and high-dose ICS (>800 µg/day). In the primary analysis, ICS exposure was treated as a time-fixed variable.

The primary outcome was the first detection of NTM from lower respiratory tract samples. We included all positive NTM samples whether they were from sputum, bronchoalveolar lavage, pleurocentesis, or lung biopsy. Radiographic and clinical criteria were not applied due to lack of nationwide data. In this article, we refer to all cases as NTM isolation to stay in consensus with guidelines [23]. However, the distribution of clinical significance was investigated to indicate whether cases had isolation or pulmonary disease. Clinical significance was measured based on number of positive cultures and sample source, using a previously validated method [24,25]; see Supplementary Table S3. Follow-up ended at the earliest occurrence of NTM isolation, death, or end of follow-up at 31 December 2022.

### 2.3. Statistical Analysis

Hazards of NTM isolation associated with ICS treatment were estimated using a cause-specific Cox proportional hazards regression models with death as a competing risk. The model was adjusted for possible confounders: entry year in quartiles (2008–2009, 2010–2012, 2013–2016, 2017–2021), age at entry in quartiles (30–62, 63–71, 72–78, 79–108), sex (male, female), BMI (<18.5 kg/m^2^, 18.5–25 kg/m^2^, >25 kg/m^2^), tobacco exposure (never, former, current smoker), FEV1% (>80%, 50–79%, 30–49%, <30%), Charlson comorbidity index (CCI) (≤2, 3–4, ≥5), presence of bronchiectasis (DJ47), exacerbation status, and use of oral corticosteroids (OCS). Exacerbation status was determined in regard to whether patients had a exacerbation requiring hospital admission in the year preceding the index date. Exacerbations were evaluated using ICD10 codes DJ440, DJ441, and DJ449, only including hospitals stays exceeding 1 day. The OCS dose was calculated as a yearly cumulative dose and was based on redeemed prescriptions in the year prior to the index date. Patients were divided into three groups: no OCS, low-dose (below the median, <750 mg/year), and high-dose (above the median, ≥750 mg/year).

For patients where data on BMI were unavailable, but both height and weight were registered; these were used to calculate BMI. If FEV1% was unavailable, but age, height, and FEV1 in liters were recorded, the predicted FEV1 in liters was calculated, and FEV1% were derived accordingly. Variables were excluded if they were physiologically impossible, e.g., BMI < 6 kg/m^2^ or >75 kg/m^2^ and FEV1 < 5% or >165%. Smokers currently undergoing smoking cessation were categorized as current smokers. To illustrate the causal relationship, a directed acyclic graph was constructed; see Supplementary Figure S1.

Cumulative incidence of NTM isolation was calculated across exposure groups. Sensitivity analyses included a complete-case analysis and an inverse probability of treatment weighted (IPTW) Cox regression analysis, with balances evaluated using standardized mean differences (SMDs), aiming for SMDs < 0.1 [26]. We investigated whether patients remained in the same ICS group throughout follow-up and performed a time-dependent Cox regression analysis with yearly updated exposure. Additionally, an unadjusted and adjusted Cox regression model only including patients with definite and possible disease were performed. Analyses were performed using R (version 4.4.3), using packages mice, ggplot2, survival, and twang [27,28,29,30]. Statistical significance was defined as *p*-values < 0.05. Hazard ratios (HRs) with 95% confidence intervals (95% CIs) were reported. Missing variables were handled with multiple imputation. The assumption of proportional hazards was evaluated using a proportionality test and Schoenfeld residuals.

### 2.4. Ethics

In accordance with Danish law, the authors were granted access to data in the nationwide registers (Data Protection Agency: 2012-58-0004) without the requirement for ethical approval or individual patient consent.

## 3. Results

**Figure 1:**
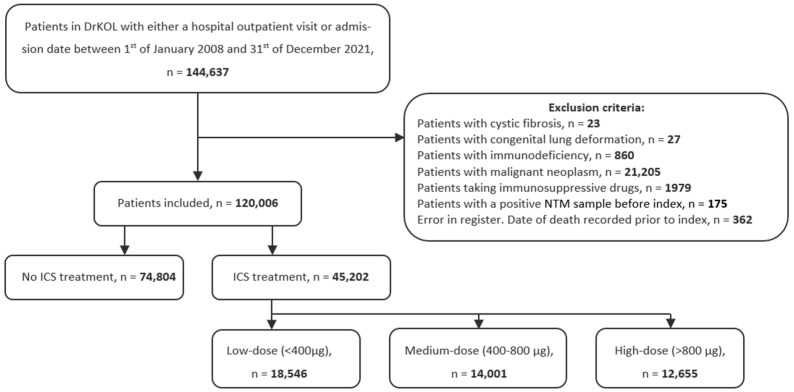
Study population before and after exclusion. We excluded patients with a diagnosis of cystic fibrosis, congenital lung malformation, immunodeficiency, or malignant neoplasm within 5 years before cohort entry. We excluded patients with at least one prescription of any systemic immunosuppressive drug in the year prior to the index date. ICS treatment was based on redeemed prescription in the year prior to the index date and calculated as the budesonide-equivalent dose. Patients were categorized as non-users, low-dose (<400 µg/day), medium-dose (400–800 µg/day), and high-dose (>800 µg/day).

We identified 144,637 patients in the DrCOPD register with an admission date or hospital outpatient visit between 2008 and 2021. We excluded 24,631 of these patients; see Figure 1. The majority of these were excluded due to a cancer diagnosis or treatment with systemic immunosuppressive drugs. Due to an error in the register, we had to exclude 362 patients, as they were recorded as having a date of death before their inclusion date in DrCOPD. In total, we included 120,006 patients; whereof 45,202 (37.7%) patients received ICS treatment in the year prior to the index date. In the entire DrCOPD population, 831 cases had NTM isolated from the respiratory sample, of whom 469 were excluded, most commonly due to isolation of NTM outside the study period or due to a cancer diagnosis.

Baseline characteristics are shown in Table 1. ICS users had more airway obstruction, and a greater use of OCS compared to non-ICS users. Over a median follow-up time of 4.9 years, 378 (0.32%) patients met the primary outcome of a positive NTM culture, and 61,734 (51.4%) patients died.

### 3.1. Main Analyses

In the unadjusted Cox regression analysis, medium- and high-dose ICS treatment were associated with an increased hazard of NTM isolation compared to no ICS treatment. The association was dose-dependent and remained significant after adjusting for potential confounders (Table 2). The assumption of proportional hazards was evaluated and showed no significant violation.

Increasing age was associated with a decreased hazard of NTM isolation in the age group 72–78 years and 79–106 years compared with the age group 30–62 (HR 0.59, 95% CI 0.43–0.81, *p*-value = 0.001; and HR 0.38, 95% CI 0.25–0.57, *p*-value < 0.001, respectively). Females had a lower hazard than males (HR 0.78, 95% CI 0.63–0.96, *p*-value = 0.020). A higher degree of airway obstruction was associated with an increased hazard for NTM isolation for FEV1 30–49% and FEV1 < 30% compared with FEV1 ≥ 80%, (HR 1.99, 95% CI 1.02–3.89, *p*-value = 0.043; and HR 2.79, 95% CI 1.39–5.58, *p*-value = 0.004, respectively). A high BMI was associated with a decreased hazard (HR 0.49, 95% CI 0,38–0.63, *p*-value < 0.001), while a low BMI was associated with an increased hazard (HR 1.65, 95% CI 1.21–2.26, *p*-value = 0.002) compared with a BMI of 18.5–25 kg/m^2^. Prescence of bronchiectasis was associated with an increased hazard (HR 2.07, 95% CI 1.09–3.90, *p*-value = 0.026). A cumulative dose of OCS over 750 mg in the year before index was associated with an increased hazard (HR 1.82, 95% CI 1.41–2.35, *p*-value < 0.001) compared to no OCS. Index year, smoking status, CCI, and having a hospital visit required due to exacerbation in the year before the index date were not significantly associated with the hazard of NTM isolation. Results for all included variables are shown in Table 3.

**Figure 2:**
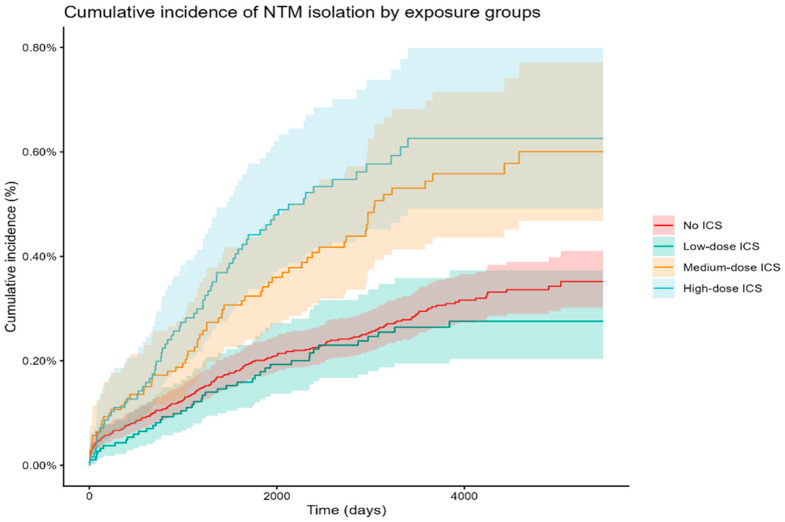
Cumulative incidence of NTM isolation in the four treatment groups: no ICS, low-dose (<400 µg/day), medium-dose (400–800 µg/day), and high-dose (>800 µg/day). Treatment groups were defined using ICS prescriptions in the year prior to the index date and calculated as mean daily budesonide equivalent dose.

Figure 2 shows the cumulative incidence of NTM isolation in the four treatment groups: no ICS, low-dose, medium-dose, and high-dose. The incidence of NTM isolation in the treatment group with medium- or high-dose ICS was higher compared with non-ICS and low-dose. The NTM isolation incidence for low-dose ICS was not elevated compared with no ICS. The most prevalent species were MAC (69.6%) and *M. xenopi* group (9.5%); see Supplementary Figure S2 for a complete overview of isolated NTM species. We investigated the distribution of clinical significance and found a larger percentage of patients with definite disease in the high-dose ICS group compared to the rest; see Supplementary Table S4.

### 3.2. Sensitivity Analyses

We conducted a complete-case analysis and an IPTW Cox regression analysis, achieving SMD < 0.1. In both analyses, the association between high-dose ICS and the hazard of NTM isolation remained significant, whereas the association observed for medium-dose ICS was no longer statistically significant (Table 4).

We investigated whether patients stayed in the same treatment group during the entire study period and found that, on average, 19.2% patients changed treatment groups each year. Therefore, we performed an unadjusted and adjusted time-dependent Cox regression analysis. In both analyses, the association between high-dose ICS and NTM isolation remained significant, while the association between medium-dose ICS and NTM isolation was no longer significant (Table 5). Results for all included variables in the adjusted analysis are shown in Supplementary Table S5.

We investigated whether results remained robust, if only including patients with definite and possible disease in an unadjusted and adjusted Cox regression analysis (Table 6). The association between medium- and high-dose ICS and hazard of NTM isolation was significant in the unadjusted analysis and remained significant for high-dose ICS in the adjusted analysis.

## 4. Discussion

This national register-based cohort study included more than 120,000 patients with COPD and demonstrated an increased hazard of NTM isolation associated with medium- and high-dose ICS treatment. This association was dose-dependent and remained significant for high-dose ICS use across multiple sensitivity analyses, including a time-dependent Cox regression analysis, indicating a robust and consistent signal.

Yu et al. examined the association between ICS treatment and the risk of NTM-PD in a propensity score-matched cohort of over 90,000 patients with COPD from South Korea and reported results that closely resembled ours [15]. Likewise, Shu et al. conducted a meta-analysis of available case-control studies and found a dose-dependent relationship between ICS and NTM-PD, with a 2-fold increased risk among patients who had received ICS treatment within the year before NTM-PD diagnosis [14]. Notably, Andréjak et al. performed a Danish case-control study and reported an odds ratio of 29 for patients with COPD currently using ICS [13]. Although they also found a dose-dependent relationship, their results differ substantially from ours. This discrepancy may be explained by different study designs and methods: Andréjak et al. [13] matched only for age, sex, and place of residence but not disease, increasing the likelihood of residual confounding. Furthermore, the analysis was only adjusted for comorbidity and alcoholism-related conditions, in contrast to the present study which adjusted for several possible confounders.

Those studies investigated NTM-PD, while this study investigated NTM isolation. While only 25% of patients in this study were categorized as isolations, the rest were categorized as definite or possible disease. Earlier validations show that 95.8–100% of patients with definite disease and 73.3–90% of patients with possible disease met diagnostic criteria of NTM-PD [24,25]. Therefore, this study is more likely to reflect the hazard of NTM-PD rather than just NTM isolation. Overall, our findings support and strengthen the evidence of a dose-dependent association between ICS treatment and the risk of NTM-PD in patients with COPD.

COPD itself is a known risk factor for pneumonia due to chronic bronchitis, increased mucus production, and structural airway changes [31]. Several mechanisms have been proposed to explain how ICS may further increase this risk. ICS is thought to alter lung macrophage function, an essential component of the innate immune system, thereby impairing the clearance of inhaled pathogens [32]. A narrative review has also suggested that ICS treatment of COPD with a neutrophilic endotype may alter the lung microbiome and increase bacterial load, potentially explaining the increased risk of pneumonia [33]. A dose-dependent association between ICS use and other respiratory pathogens have been demonstrated previously. For instance, ICS use, particularly at high doses, has been associated with an increased risk of *Mycobacterium tuberculosis*, *Pseudomonas aeruginosa*, and *Streptococcus pneumoniae* [34,35,36]. Our results are consistent with these findings, demonstrating a similar dose-dependent relationship between ICS use and risk of NTM isolation.

This study has several strengths. We had access to comprehensive nationwide registers and could link patient-level data across multiple datasets through Denmark’s unique personal identification system. The cohort was large and well-characterized, with all patients having a specialist-verified diagnosis of COPD recorded in the DrCOPD registry. The availability of detailed clinical data enabled adjustment for several important confounders and markers of disease severity. Many of these variables are risk factors for both NTM and *Mycobacterium tuberculosis*, such as age, sex, and BMI. Having the opportunity to adjust for this strength, the robustness of the analyses and results of this study align well with results from studies of *M. tuberculosis*, finding that a low BMI is a risk factor for infection, while a high BMI is protective [37,38].

However, several limitations should also be acknowledged. First, as with all register-based studies, there is a risk of misclassification bias. ICS exposure was inferred from prescription data, which does not account for non-adherence. Patients could also initiate, discontinue, or modify ICS treatment during the follow-up; we addressed this limitation by including a time-dependent cox regression model as a sensitivity analysis. Although recommendations for ICS changed during the study period, the exposure groups were defined based on budesonide-equivalent doses. Patients were, therefore, categorized according to exposure, regardless of the treatment guideline in place at the time. Furthermore, analyses were adjusted for the index year, and a time-dependent Cox regression analysis was performed, taking changing ICS doses into account. Second, some variables were missing in the DrCOPD registry, particularly for BMI, smoking status, and grade of obstruction. We addressed this using multiple imputations in the main analysis, as well as conducting a complete-case analysis to assess the robustness of our results. Third, the data in DrCOPD were derived from many different hospitals across Denmark, which may introduce observer bias if data collection procedures were not entirely uniform. To minimize this risk, we excluded physiological implausible values (e.g., BMI < 6 kg/m^2^ and FEV1 < 5%). Fourth, increasing age was associated with a lower hazard of NTM isolation in our study, which contrasts with previous literature showing a higher prevalence of NTM-PD among older individuals. This finding should, therefore, be interpreted with caution. Several factors may explain this discrepancy. First, older and more frail patients may be less likely to undergo diagnostic evaluation for NTM, leading to underdetection. Second, competing risk of death may influence the results, as older patients have a higher mortality rate and may die before NTM is identified. Third, selection bias may be present, as inclusion in the cohort required specialist care, potentially resulting in a relatively healthier subgroup of elderly patients. Overall, these factors may contribute to an underestimation of NTM risk in the oldest age groups in this study. Fifth, low-dose ICS was associated with a hazard ratio below 1, although this finding was not statistically significant. This could suggest a potential protective effect; however, we find this unlikely. A more plausible explanation is residual confounding or confounding by indication. Patients prescribed low-dose ICS may represent a subgroup with milder disease, fewer exacerbations, or better overall health status, which may be associated with a lower underlying risk of NTM isolation. Although we adjusted for multiple markers of disease severity, including FEV1, exacerbation history, and comorbidity burden, residual confounding cannot be excluded. Therefore, this finding should be interpreted with caution and not as evidence of a protective effect. Whether similar findings would be observed in other European registries is uncertain, as this may depend on differences in prescribing patterns, disease severity distributions, and diagnostic practices. Finally, we were not able to evaluate radiographic or clinical criteria to confirm NTM-PD diagnoses. This is a limitation, as microbiological isolation does not necessarily reflect clinically significant diseases and may lead to an overestimation of the disease burden. To account for this, we evaluated clinical significance. Based on clinical significance, only 25% of cases had NTM isolation; the rest had definite or possible disease. A Cox regression only including patients with definite and possible disease was performed and showed that the results were robust with regard to the association between NTM-PD and use of ICS.

## 5. Conclusions

In conclusion, we found that high-dose ICS use in patients with COPD was associated with a 1.5-fold increased hazard of NTM isolation compared with non-ICS use. Clinicians should consider this risk when prescribing high-dose ICS, particularly in patients with other predisposing factors for mycobacterial disease. Our findings reinforce existing evidence for a dose-dependent relationship between ICS treatment and NTM isolation in patients with COPD. Prospective studies are warranted to establish causality, though conducting such trials would be challenging, given the relatively low incidence of NTM isolation.

## Figures and Tables

**Table 1:** Baseline characteristics for patients with COPD. Data are presented as n (%) or median (IQR). Patients were stratified by ICS according to the budesonide-equivalent dose used in the year prior to the index date and categorized as non-users, low-dose (<400 µg/day), medium-dose (400–800 µg/day), and high-dose (>800 µg/day). ICS: inhaled corticosteroid, NTM: non-tuberculous mycobacteria, BMI: body mass index, CCI: Charlson comorbidity index. OCS: oral corticosteroid.

		No ICS n = 74,804	Low-Dose n = 18,546	Medium-Dose n = 14,001	High-Dose n = 12,655
ICS, median (IQR)	Mean daily dose, µg/day	0 (0)	197 (105–286)	578 (473–657)	1262 (946–1682)
NTM isolation, n (%)	Cases	200 (0.27)	43 (0.23)	67 (0.48)	68 (0.54)
Age, n (%)	30–62	19,105 (25.5)	5615 (30.3)	3269 (23.3)	3287 (26.0)
	63–71	18,940 (25.3)	4790 (25.8)	4011 (28.6)	3768 (29.8)
	72–78	16,782 (22.4)	4092 (22.1)	3558 (25.4)	2961 (23.4)
	79–106	19,977 (26.7)	4049 (21.8)	3163 (22.6)	2639 (20.9)
Sex, n (%)	Female	38,442 (51.4)	9929 (53.5)	7584 (54.2)	7286 (57.6)
Index year, n (%)	2008–2009	21,039 (28.1)	6174 (33.3)	5091 (36.4)	4106 (32.4)
	2010–2012	16,765 (22.4)	4548 (24.5)	3092 (22.1)	2101 (16.6)
	2013–2016	21,388 (28.6)	3927 (21.2)	3040 (21.7)	4386 (34.7)
	2017–2021	15,612 (20.9)	3897 (21.0)	2778 (19.8)	2062 (16.3)
Grade of obstruction, n (%)	FEV1 ≥ 80%	2884 (8.6)	720 (6.9)	324 (4.0)	247 (3.4)
	FEV1 50–79%	15,515 (46.3)	4856 (46.3)	2861 (35.6)	2294 (31.4)
	FEV1 30–49%	11,221 (33.5)	3738 (35.6)	3389 (42.2)	3137 (43.0)
	FEV1 < 30%	3872 (11.6)	1180 (11.2)	1461 (18.2)	1622 (22.2)
BMI, n (%)	<18.5 kg/m^2^	3207 (9.3)	810 (7.6)	771 (9.4)	806 (10.8)
	18.5–25 kg/m^2^	12,826 (37.3)	3881 (36.2)	3199 (38.9)	2890 (38.7)
	>25 kg/m^2^	18,399 (53.4)	6032 (56.3)	4250 (51.7)	3776 (50.5)
Smoking status, n (%)	Never smoker	1154 (3.4)	493 (4.6)	301 (3.7)	292 (3.9)
	Former smoker	17,897 (52.4)	5927 (55.4)	5170 (63.0)	4367 (58.9)
	Current smoker	15,128 (44.3)	4271 (39.9)	2736 (33.3)	2751 (37.1)
CCI n (%)	CCI ≤ 2	59,638 (79.7)	15,180 (81.9)	11,675 (83.4)	10,714 (84.7)
	CCI 3–4	10,680 (14.3)	2457 (13.2)	1731 (12.4)	1492 (11.8)
	CCI ≥ 5	4486 (6.0)	909 (4.9)	595 (4.2)	449 (3.5)
Bronchiectasis (%)	Yes	849 (1.1)	279 (1.5)	172 (1.2)	184 (1.5)
Exacerbation (%)	Yes	22,817 (30.5)	7423 (40.0)	6258 (44.7)	5537 (43.8)
Cumulative OCS use in the year before index, n (%)	No OCS	57,905 (77.4)	11,745 (63.3)	7776 (55.5)	6091 (48.1)
	<750 mg/year	8792 (11.8)	3730 (20.1)	2917 (20.8)	2666 (21.1)
	≥750 mg/year	8107 (10.8)	3071 (16.6)	3308 (23.6)	3898 (30.8)

**Table 2:** Unadjusted and adjusted Cox proportional hazards regression model. The adjusted model included age in quartiles, sex, index year in quartiles, FEV1%, BMI, smoking status, CCI, presence of bronchiectasis, exacerbation status, and mean daily OCS use during the year before index. The hazards indicate the risk of NTM isolation at any time during the follow-up period, accounting for death as a competing risk. Asterisks indicate statistical significance: * = *p* < 0.05, ** = *p* < 0.01, *** = *p* < 0.001.

	Unadjusted HR (95% CI)	*p*-Value	Adjusted HR (95% CI)	*p*-Value
No ICS	1.00	Ref.	1.00	Ref.
Low-dose ICS	0.79 (0.57–1.10)	0.163	0.74 (0.53–1.03)	0.076
Medium-dose ICS	1.71 (1.30–2.26)	<0.001 ***	1.39 (1.04–1.86)	0.024 *
High-dose ICS	2.01 (1.53–2.65)	<0.001 ***	1.51 (1.13–2.02)	0.006 **

**Table 3:** Variables of the adjusted Cox proportional hazards regression model. The hazards indicate the risk of NTM isolation at any time during the follow-up period, accounting for death as a competing risk. Asterisks indicate statistical significance: * = *p* < 0.05, ** = *p* < 0.01, *** = *p* < 0.001.

	Adjusted HR (95% CI)	*p*-Value
Sex: male	1.00	Ref.
Sex: female	0.78 (0.63–0.96)	0.020 *
Age, 1st quartile	1.00	Ref.
Age, 2nd quartile	1.09 (0.86–1.39)	0.472
Age, 3rd quartile	0.59 (0.43–0.81)	0.001 **
Age, 4th quartile	0.38 (0.25–0.57)	<0.001 ***
Index year, 1st quartile	1.00	Ref.
Index year, 2nd quartile	1.13 (0.88–1.45)	0.354
Index year, 3rd quartile	0.80 (0.60–1.07)	0.137
Index year, 4th quartile	0.76 (0.50–1.16)	0.209
FEV1 ≥ 80%	1.00	Ref.
FEV1 50–79%	1.75 (0.87–3.50)	0.112
FEV1 30–49%	1.99 (1.02–3.89)	0.043 *
FEV1 < 30%	2.79 (1.39–5.58)	0.004 **
BMI 18.5–25 kg/m^2^	1.00	Ref.
BMI < 18.5 kg/m^2^	1.65 (1.21–2.26)	0.002 **
BMI > 25 kg/m^2^	0.49 (0.38–0.63)	<0.001 ***
Non-smokers	1.00	Ref.
Former smokers	1.19 (0.56–2.51)	0.649
Current smokers	1.19 (0.57–2.48)	0.647
CCI ≤ 2	1.00	Ref.
CCI: 3–4	0.78 (0.54–1.14)	0.203
CCI ≥ 5	0.76 (0.39–1.48)	0.413
No bronchiectasis	1.00	Ref.
Bronchiectasis	2.07 (1.09–3.90)	0.026 **
No hospital requiring exacerbation	1.00	Ref.
≥1 hospital requiring exacerbation	1.12 (0.91–1.38)	0.300
No OCS	1.00	Ref.
OCS < 750 mg/year	1.08 (0.80–1.45)	0.629
OCS ≥ 750 mg/year	1.82 (1.41–2.35)	<0.001 ***

**Table 4:** Complete-case analysis using Cox regression analysis, adjusted for age in quartiles, sex, index year in quartiles, FEV1%, BMI, smoking status, CCI, presence of bronchiectasis, exacerbation status, and mean daily OCS use during the year before index. IPTW analysis with achieved SMD < 0.1. The hazards indicate the risk of NTM isolation at any time during the follow-up period, accounting for death as a competing risk. Asterisks indicate statistical significance: * = *p* < 0.05.

	Complete Case HR (95% CI)	*p*-Value	IPTW HR (95% CI)	*p*-Value
No ICS	1.00	Ref.	1.00	Ref.
Low-dose ICS	0.71 (0.48–1.05)	0.088	0.70 (0.47–1.04)	0.081
Medium-dose ICS	1.31 (0.94–1.83)	0.112	1.35 (0.96–1.90)	0.084
High-dose ICS	1.45 (1.04–2.03)	0.030 *	1.50 (1.03–2.17)	0.034 *

**Table 5:** Unadjusted and adjusted time-dependent Cox regression model. The adjusted model included age in quartiles, sex, index year in quartiles, FEV1%, BMI, smoking status, CCI, presence of bronchiectasis, exacerbation status, and mean daily OCS use during the year before index. The hazards indicate the risk of NTM isolation at any time during the follow-up period, accounting for death as a competing risk. Asterisks indicate statistical significance: *** = *p* < 0.001.

	Unadjusted HR (95% CI)	*p*-Value	Adjusted HR (95% CI)	*p*-Value
No ICS	1.00	Ref.	1.00	Ref.
Low-dose ICS	1.16 (0.83–1.63)	0.372	1.10 (0.78–1.54)	0.592
Medium-dose ICS	1.31 (0.96–1.80)	0.088	1.14 (0.83–1.57)	0.412
High-dose ICS	2.35 (1.83–3.02)	<0.001 ***	1.92 (1.48–2.49)	<0.001 ***

**Table 6:** Unadjusted and adjusted Cox regression analysis only including patients with definite or possible disease. The adjusted model included age in quartiles, sex, index year in quartiles, FEV1%, BMI, smoking status, CCI, presence of bronchiectasis, exacerbation status, and mean daily OCS use during the year before index. The hazards indicate the risk of NTM isolation at any time during the follow-up period, accounting for death as a competing risk. Asterisks indicate statistical significance: * = *p* < 0.05, ** = *p* < 0.01, *** = *p* < 0.001.

	Unadjusted HR (95% CI)	*p*-Value	Adjusted HR (95% CI)	*p*-Value
No ICS	1.00	Ref.	1.00	Ref.
Low-dose ICS	0.75 (0.51–1.10)	0.140	0.68 (0.46–1.00)	0.052
Medium-dose ICS	1.60 (1.16–2.22)	<0.004 **	1.26 (0.90–1.76)	0.182
High-dose ICS	2.04 (1.49–2.79)	<0.001 ***	1.44 (1.03–2.01)	0.031 *

## Data Availability

Restrictions apply to the availability of these data. Data were obtained from the Danish National Health Authority and are available at https://sundhedsdatastyrelsen.dk/da/forskerservice/ansog-om-data (accessed on 23 April 2026) with the permission of the Danish National Health Authority.

## Supplementary Materials

The following supporting information can be downloaded at: https://www.mdpi.com/article/10.3390/jcm15093352/s1, Table S1: Sample size calculation; Table S2: Budesonide equivalent conversion ratios; Table S3: Clinical significance definition; Table S4: Distribution of clinical significance; Table S5: Time-dependent cause-specific Cox proportional hazard regression results with adjusted variables; Figure S1: Directed acyclic graph; Figure S2: Distribution of NTM species.

## Abbreviations

The following abbreviations are used in this manuscript:

BMI	Body mass index
CCI	Charlson comorbidity index
COPD	Directory of open access journals
DrCOPD	The Danish Register of Chronic Obstructive Pulmonary Disease
HR	Hazard ratio
ICS	Inhaled corticosteroid
IPTW	Inverse probability of treatment weighted model
MAC	*Mycobacterium avium* complex
NTM	Non-tuberculous mycobacterial pulmonary disease
OCS	Oral corticosteroid
SMD	Standardized mean differences
95% CI	95% confidence interval
