# Supplementary material for: Inhaled Corticosteroids and Non-Tuberculous Mycobacteria Risk in Patients with COPD
Source: J Clin Med. 2026 Apr 28;15(9):3352. doi: 10.3390/jcm15093352 (PMC13163958; doi:10.3390/jcm15093352)
Supplement: Supplementary file 1 [file jcm-15-03352-s001.zip › jcm-4245798-supplementary.pdf]

Supplementary

Supplementary
- Supplementary Table S1: Sample size calculation
- Supplementary Table S2: Budesonide equivalent conversion ratios
- Supplementary Table S3: Clinical significance definition
- Supplementary Table S4: Distribution of clinical significance
- Supplementary Table S5: Time-dependent cause-specific Cox proportional hazard regression results with adjusted variables
- Supplementary Figure S1: Directed acyclic graph
- Supplementary Figure S2: Distribution of NTM species

Supplementary Table S1: Sample size calculation

**Sample size calculation**
Assuming 1% of patient who doesn't get ICS treatment meet the primary outcome within the follow up period and hypothesizing a 5% absolute risk reduction or increase (2-sided comparison) while accepting a risk of a type 1 error limit (α) of 5% and a risk of a type 2 error of maximum 20% (corresponding to a power of at least 80%). The aim of this study is to have a sample size of 8150 per group.

Supplementary Table S2: Budesonide equivalent conversion ratios

**ICS type**	**Budesonide equivalence conversion ratio**
Beclomethasone	1:1
Mometasone	1:1
Beclomethasone HFA	1:2
Fluticasone	1:2
Ciclesonide	1:2.5
Fluticasone furoate	1:10

Supplementary Table S3: Clinical significance definition

**Clinical significance, based number of positive NTM cultures and sample source**	
Definite disease	>3 positive NTM cultures with the same species, or ≥3 positive cultures, including at least one obtained by bronchoscopy or pleurocentesis, or ≥1 positive culture from a lung biopsy

Isolation of NTM	Only 1 positive culture of NTM
Possible disease	The remaining cases

Supplementary Table S4: Distribution of clinical significance

	**Incidence of isolation of NTM in treatment groups**		**Distribution of clinical significance**		
	**Population, n (%)**	**Cases, n (%)**	**Definite disease, n (%)**	**Possible disease, n (%)**	**Isolation, n (%)**
**Total**	120006 (100)	378 (0.32)	115 (30.4)	170 (45.0)	93 (24.6)
**No ICS**	74804 (62.3)	200 (0.27)	58 (29.0)	95 (47.5)	47 (23.5)
**Low-dose ICS**	18546 (15.5)	43 (0.23)	12 (27.9)	19 (44.2)	12 (27.9)
**Medium-dose ICS**	14001 (11.7)	67 (0.48)	16 (23.9)	32 (47.8)	19 (28.4)
**High-dose ICS**	12655 (11.4)	68 (0.54)	29 (42.6)	24 (35.3)	15 (22.1)

Supplementary Table S5: Time-dependent cause-specific Cox proportional hazard regression results with adjusted variables

	**HR**	**95% CI**	**P value**
No ICS	1.00	Ref.	Ref.
Low-dose ICS	1.10	0.78-1.54	0.592
Medium-dose ICS	1.14	0.83-1.57	0.412
High-dose ICS	1.92	1.48-2.49	<0.001***
Sex: male	1.00	Ref.	Ref.
Sex: female	0.79	0.64-0.97	0.027*
Age, 1st quartile	1.00	Ref.	Ref.
Age, 2nd quartile	1.09	0.86-1.39	0.466
Age, 3rd quartile	0.60	0.44-0.83	0.002**
Age, 4th quartile	0.39	0.26-0.58	<0.001***
Index year, 1st quartile	1.00	Ref.	Ref.

Index year, 2nd quartile	1.08	0.84-1.39	0.560
Index year, 3rd quartile	0.77	0.57-1.03	0.073
Index year, 4th quartile	0.72	0.48-1.10	0.131
FEV1 ≥80%	1.00	Ref.	Ref.
FEV1 50-79%	1.57	0.70-3.55	0.261
FEV1 30-49%	1.70	0.73-3.95	0.200
FEV1 < 30%	2.23	0.91-5.46	0.077
BMI 18.5 – 25 kg/m^2	1.00	Ref.	Ref.
BMI < 18.5 kg/m^2	1.49	1.10-2.02	0.01*
BMI > 25 kg/m^2	0.54	0.37-0.77	0.002**
Non-smokers	1.00	Ref.	Ref.
Former smokers	1.07	0.52-2.30	0.865
Current smokers	1.10	0.50-2.42	0.806
CCI ≤ 2	1.00	Ref.	Ref.
CCI: 3-4	0.76	0.52-1.11	0.158
CCI ≥ 5	0.75	0.38-1.47	0.401
No bronchiectasis	1.00	Ref.	Ref.
Bronchiectasis	1.99	1.05-3.78	0.034*
No hospital requiring exacerbation	1.00	Ref.	Ref.
≥1 hospital requiring exacerbation	1.14	0.92-1.40	0.236
No OCS	1.00	Ref.	Ref.
OCS < 750 mg/year	1.08	0.80-1.45	0.607
OCS ≥ 750 mg/year	1.88	1.46-2.41	<0.001***

Supplementary Figure S1: Directed acyclic graph

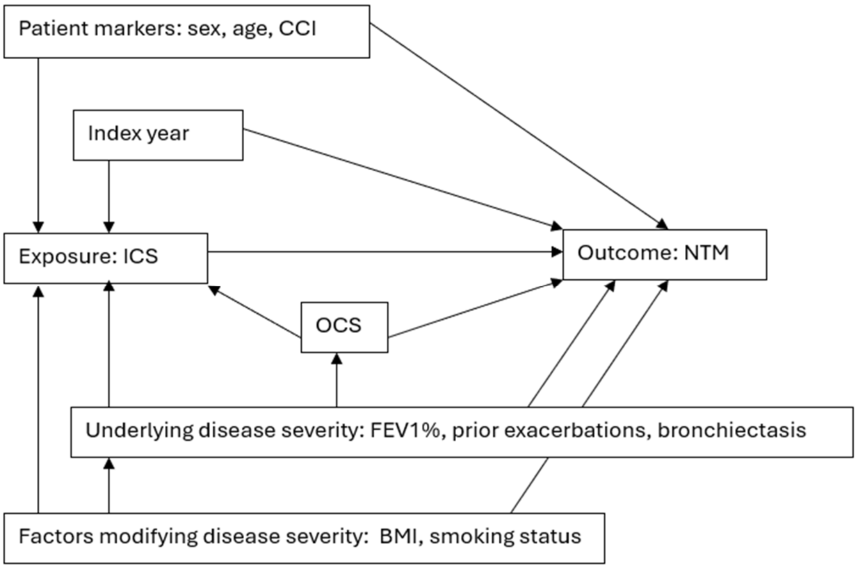

Directed acyclic graph showing the causal relationship between variables. ICS: inhaled corticosteroid, NTM: non-tuberculous mycobacteria, BMI: body mass index, CCI: Charlson Comorbidity Index. OCS: oral corticosteroid.

Supplementary Figure S2: Distribution of NTM species

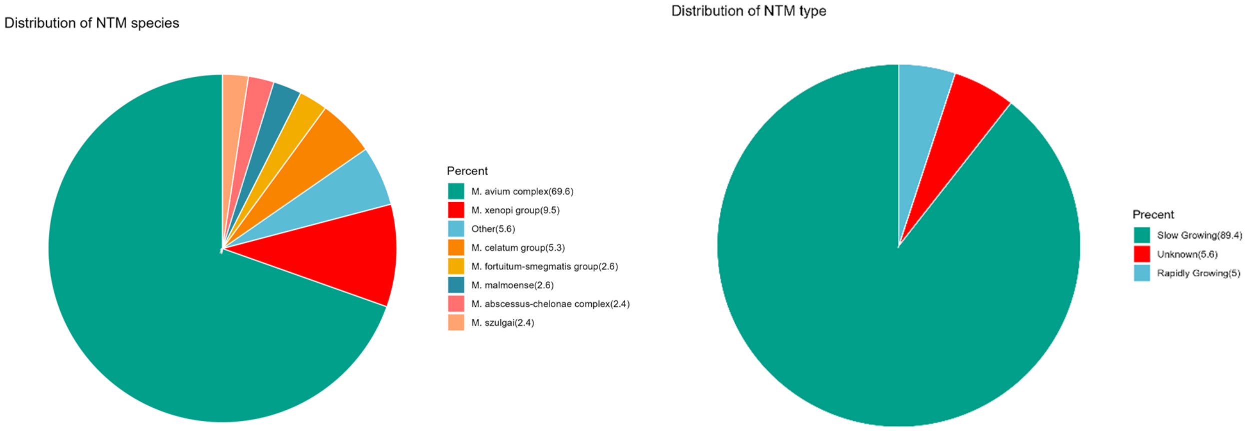

Overview of isolated NTM species in patients. Rapidly growing species: *M. fortuitum-smegmatis* group, *M. abscessus-chelonea* complex. The rest is slow growing, apart from other which is unknown.
